# Mindfulness-Based Cognitive Therapy Versus Pure Cognitive Behavioural Self-Help for Perfectionism: a Pilot Randomised Study

**DOI:** 10.1007/s12671-017-0817-8

**Published:** 2017-10-13

**Authors:** Kirsty James, Katharine A. Rimes

**Affiliations:** 1 Department of Psychology, University of Bath, Bath, BA2 7AY UK; 2 Department of Psychology, Institute of Psychiatry, Psychology and Neuroscience, King’s College London, De Crespigny Park, London, SE5 8AF UK

**Keywords:** Perfectionism, Mindfulness, Self-compassion, Students, Cognitive behavioural treatment, Self-help

## Abstract

This pilot study compared mindfulness-based cognitive therapy (MBCT) with a self-help guide based on cognitive behaviour therapy (CBT) for university students experiencing difficulties due to perfectionism. Participants were randomised to an MBCT intervention specifically tailored for perfectionism or pure CBT self-help. Questionnaires were completed at baseline, 8 weeks later (corresponding to the end of MBCT) and at 10-week follow-up. Post-intervention intention-to-treat (ITT) analyses identified that MBCT participants (*n* = 28) had significantly lower levels of perfectionism and stress than self-help participants (*n* = 32). There was significant MBCT superiority for changes in unhelpful beliefs about emotions, rumination, mindfulness, self-compassion and decentering. At 10-week follow-up, effects were maintained in the MBCT group, and analyses showed superior MBCT outcomes for perfectionism and daily impairment caused by perfectionism. Pre-post changes in self-compassion significantly mediated the group differences in pre-post changes in clinical perfectionism. Greater frequency of mindfulness practice was associated with larger improvements in self-compassion. MBCT is a promising intervention for perfectionist students, which may result in larger improvements than pure CBT self-help. The findings require replication with a larger sample.

**Electronic supplementary material:**

The online version of this article (10.1007/s12671-017-0817-8) contains supplementary material, which is available to authorized users.

## Introduction

Perfectionism has long been considered to be linked to psychological distress, with evidence that it can act as a risk or maintaining factor across psychological difficulties (Egan et al. 2011). Research with students has found that nearly two thirds can be categorised as perfectionists, with over a quarter considered maladaptive perfectionists (Grzegorek et al. 2004). Although striving for high standards is not usually problematic in itself (Shafran et al. 2002), unhealthy forms of perfectionism have been identified, sometimes known as ‘negative’ or ‘clinical’ perfectionism (Shafran and Mansell 2001). This is often associated with self-criticism, fear of failure and negative evaluation by the self or others, alongside higher levels of distress and behavioural impairments (Campbell and Paula 2002; Shafran et al. 2002; Slade and Owens 1998).

A recent cognitive behavioural model (Shafran et al. 2010) suggests that negatively biased thinking patterns and behaviours (e.g. checking, avoidance and procrastination) maintain unhealthy perfectionism. The model proposes that an individual’s self-evaluation being dependent on achievement leads them to hold inflexible standards about the level of performance they should achieve and frequently holding higher standards for themselves in comparison to others. The cognitive aspects suggested to maintain perfectionism include evaluation of how well rules are met in a dichotomous manner, self-statements incorporating ‘shoulds’ and ‘musts’, overgeneralising and selectively attending to the negative, while discounting the positive. Failure to meet these excessively demanding self-imposed standards is proposed to result in self-criticism and further counter-productive behaviours (e.g. list-making, over-preparing and being overly thorough) (Egan et al. 2014b). These responses are regarded as counter-productive because despite being aimed at helping the individual to prevent failure or to increase or maintain high standards, they can have unintended consequences which actually impair performance, such as causing tiredness or taking up time that could be used to address the full range of tasks at hand.

The cognitive behavioural model of perfectionism also suggests that standards that are met are subsequently re-appraised as not being demanding enough. There is increasing evidence consistent with this cognitive behavioural model, for example research suggests that self-critical thinking, dichotomous thinking and dysfunctional standards are characteristic of negative perfectionism (Egan et al. 2007; James et al. 2015). Experimental findings also suggest that perfectionism plays a role in standards being set high prior to performance (Egan et al. 2012), while findings have been mixed about standard setting following success or failure (Kobori et al. 2009; Egan et al. 2012).

This model of perfectionism also incorporates the role of processes involved in emotion regulation, such as worry and rumination in maintaining difficulties with perfectionism. Rumination is often defined as repetitive thinking about oneself, one’s problems and feelings of distress (Nolen-Hoeksema 1991). Much previous research has shown that rumination is associated with increased subsequent distress (Watkins 2008). Evidence suggests that perfectionists are more likely than others to ruminate and that rumination may mediate the relationship between maladaptive perfectionism and distress (Di Schiena et al. 2012; Short and Mazmanian 2013). Although any causal direction cannot be ascertained from cross-sectional studies, these findings are consistent with the possibility that rumination is one reason why perfectionist individuals tend to experience greater distress. Also in relation to emotional regulation, there is preliminary evidence that unhealthy perfectionism is associated with perfectionist attitudes towards emotions, in particular beliefs that negative emotions are unacceptable and can lead to negative reactions by others (Rimes and Chalder 2010). A cross-sectional study with university students found that unhelpful beliefs about emotions mediated the relationship between unhealthy perfectionism and emotional suppression; furthermore, emotional suppression mediated the relationship between unhealthy perfectionism and depressive symptomatology (Tran and Rimes 2017).

Another suggestion from the cognitive behavioural approach to perfectionism is that in line with the high levels of self-criticism, perfectionists tend to have low levels of self-compassion (Shafran et al. 2010). Consistent with this, Neff (2003) found that students high in self-compassion showed lower perfectionism. Furthermore, James et al. (2015) found that a factor on which both self-criticism and self-compassion had high loadings mediated the relationship between unhealthy perfectionism and psychological distress in a predominant student sample. Self-compassion is often seen as a key component of mindfulness and there is increasing evidence that trait mindfulness is lower in perfectionist individuals. For example, Hinterman et al. (2012) report a significant correlation between lack of mindfulness, negative perfectionism, depression and rumination. Argus and Thompson (2008) found that mindful awareness mediated the positive association between maladaptive perfectionism and depression severity.

Evidence to date suggests that psychological interventions, particularly cognitive behaviour therapy, targeting perfectionism-specific unhelpful thinking patterns and behaviours can be beneficial (Egan et al. 2014a, b; Handley et al. 2015; Lloyd et al. 2014; Pleva and Wade 2007; Riley et al. 2007; Steele et al. 2013; Steele and Wade 2008). Another form of intervention which may be helpful for perfectionism is mindfulness-based cognitive therapy (MBCT), an evidence-based treatment originally developed for depressive relapse (Teasdale et al. 2000). There is evidence that MBCT addresses processes that have been identified as important in perfectionism, as described above. For example, the effect of MBCT for recurrent depression is mediated by increases in self-compassion and mindfulness (Kuyken et al. 2010). Furthermore, mindfulness approaches successfully reduce rumination (Heeren and Philippot 2011) and unhelpful beliefs about emotions (Rimes and Chalder 2010), and increase a decentered perspective on thoughts (Teasdale et al. 2002). This raises the possibility that MBCT might be an effective alternative approach to addressing perfectionism which is associated with specific unhelpful beliefs, self-critical thinking and low self-compassion, low trait mindfulness, unhelpful beliefs about the acceptability of emotions and greater rumination.

Adapting an MBCT approach for perfectionism would have the potential advantage of being based on the cognitive behavioural model of perfectionism and drawing on the associated treatment methods (both of which are associated with accumulating supporting evidence as briefly outlined above), while also drawing on additional mindfulness methods to help address processes which may act to maintain perfectionism and associated distress. As with other mindfulness-based approaches, learning how to decenter from unhelpful thinking patterns in MBCT may be helpful for perfectionist individuals to notice perfectionism-related thoughts arising without necessarily assuming that they are true or acting on them. There have been no previous studies of MBCT for perfectionism. However, there is preliminary evidence that mindfulness-based approaches may be helpful for perfectionism from a randomised study which recruited adults experiencing distress associated with perfectionism (Wimberley et al. 2016). Compared to a wait-list group, participants allocated to reading a self-help book about mindfulness for perfectionism over a 6-week period showed greater reductions in perfectionism, negative affect and stress post-intervention, with reductions maintained at 6-week follow-up.

Also exploring self-help interventions, Steele and Wade (2008) randomised participants with bulimia nervosa to 6 weeks of guided self-help focused on either CBT for perfectionism, CBT for bulimia nervosa or a placebo condition. At post-treatment, all conditions were found to show significant improvements in symptoms of bulimia, depression and perfectionism, while at 6-month follow-up, significant differences were maintained in bulimic symptoms and perfectionism (as measured by the concern over mistakes subscale). The manual developed as the control condition drew on techniques from MBCT; however, it was not focused on perfectionism, and the authors specify that the dismantled nature of the intervention meant that it could not be classified as a mindfulness treatment (Steele and Wade 2008, p. 1317).

In this study, students experiencing perfectionism were randomly allocated to an adapted form of MBCT for clinical perfectionism or a cognitive behavioural self-help guide. This study was designed to gain information about the acceptability and feasibility of delivering a course of MBCT for perfectionist university students, and preliminary estimation of the degree of change in perfectionism associated with MBCT versus pure self-help. Pure self-help was chosen as an evidence-based minimal treatment control condition to control for the effects of receiving psycho-education and advice about the CBT approach to perfectionism; this was considered more ethical than a waiting list control condition. The primary outcome was perfectionism; secondary outcomes included impairment caused by perfectionism and self-reported depression, anxiety and stress. Exploratory analyses examined changes in self-compassion, rumination, unhelpful beliefs about emotions, mindfulness, decentering and whether these mediated group differences in changes in perfectionism.

## Method

### Participants

Participants were recruited over a 5-week period through advertisements on university campuses, websites and circular emails. The adverts sought to recruit students experiencing difficulties because of perfectionism or high standards. Inclusion criteria included (a) being a student age 18 or over, (b) a total score of 22 or above on the Concern over Mistakes subscale of the Frost Multidimensional Perfectionism Scale, as used in previous research (Steele et al. 2013) and (c) perfectionism causing significant distress or impairment in important areas of functioning, assessed via interview. If potential participants were on anti-depressant medication, this was required to have been stable for 3 months. Exclusionary criteria were current significant suicidal ideation, any current psychological treatment where perfectionism was being addressed or meeting DSM-IV diagnostic criteria for substance dependence or anorexia nervosa. Current suicidal ideation, substance dependence and anorexia were excluded due to the study not being set up to manage risks associated with these difficulties, unlike other local services (to which potential participants were provided contact details if these difficulties were present). Previous practice of mindfulness was not assessed or controlled for.

**Fig. 1:**
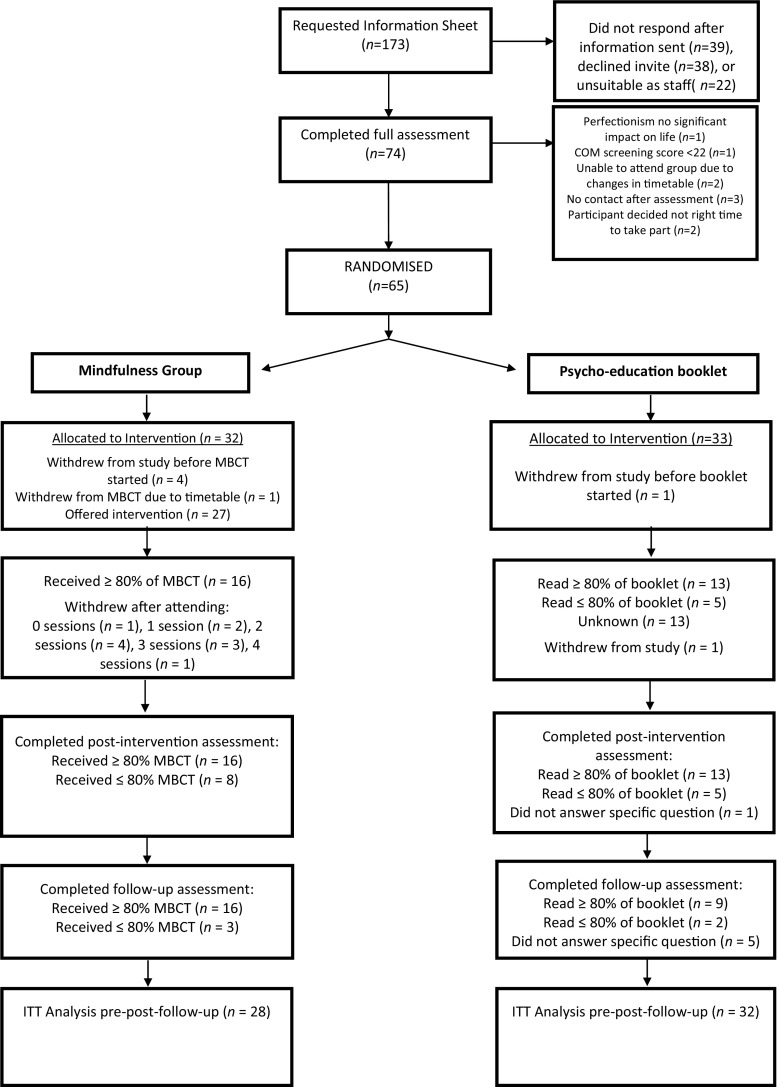
CONSORT flow chart of participant recruitment to the trial*.* MBCT mindfulness-based cognitive therapy, ITT intention-to-treat

A power analysis was not conducted as this study was aimed at investigating the acceptability and feasibility of providing an MBCT group for perfectionism in a university setting; the intention was that the preliminary outcome data gained could be used to inform subsequent power calculations. Seventy-four potential participants were assessed throughout the recruitment period of 5 weeks, which was limited by students’ term dates and to allow sufficient time for the eight MBCT sessions within this timetable. Sixty-five participants were randomised to MBCT (*n* = 32) and self-help (*n* = 33). Twenty-four MBCT and 19 self-help participants completed post-intervention assessments. Nineteen MBCT and 16 self-help participants completed follow-up assessments. The flow of participants through the trial is depicted in Fig. 1.

The sample consisted of 70% post-graduate and 30% undergraduate students. Group characteristics are presented in Table 1. Chi-square and *t* test analyses found no significant differences between the groups on any demographic variable or baseline outcome/process measure (all *p* < .05).

**Table 1:** Characteristics, engagement and perceived usefulness in MBCT and self-help samples

	MBCT (*n* = 28)		Self-help (*n* = 32)	
	Number of participants (*n*)	Percentage (%)	Number of participants (*n*)	Percentage (%)
Demographic characteristics				
Gender				
Female	23	82.1	26	81.2
Male	5	17.9	6	18.8
Age				
18–24	14	50	21	65.6
25–30	10	35.7	7	21.9
31–39	4	14.3	4	12.5
Relationship status				
Single	19	67.9	17	53.1
Partner, living abroad	1	3.5	11	34.4
Married/living together	8	28.6	4	12.5
Ethnicity				
White British	13	46.4	14	43.8
Irish	1	3.6	1	3.1
Other White background	10	35.7	7	21.9
Indian	2	7.1	2	6.2
Bangladeshi	1	3.6	0	0
Caribbean	0	0	1	3.1
African	0	0	1	3.1
Chinese	0	0	4	12.6
Other/multiracial	1	3.6	2	6.2
MINI disorders				
Generalised anxiety disorder	5	17.9	10	31.3
Major depression	3	10.7	5	15.6
Social phobia	2	7.1	4	12.5
Dysthymia	2	7.1	0	0.0
Panic disorder	1	3.6	1	3.1
Bulimia	0	0.0	1	3.1
Engagement and perceived usefulness				
Attendance/percentage of booklet read				
80% or above	16	57.1	13	40.6
50–79%	1	3.6	4	12.5
30–49%	3	10.7	1	3.1
0–29%	7	25.0	0	0.0
Did not answer/data unavailable	1	3.6	14	43.8
Perceived usefulness				
Very useful	9	32.1	0	0.0
Moderately useful	3	10.7	4	12.5
Useful	7	25.0	6	18.8
Quite useful	3	10.7	7	21.9
No use at all	1	3.6	0	0.0
Did not answer/data unavailable	5	17.9	15	46.9

### Procedure

#### Design

A pilot randomised study was undertaken, with participants randomised to MBCT (*n* = 32) or self-help (*n* = 33). Participants were assessed pre-intervention, immediately following the 8-week intervention and 10-week post-intervention.

The study protocol received approval from the Department of Psychology, University of Bath (reference: 12-124) and King’s College London Psychiatry, Nursing and Midwifery Research Ethics Subcommittee (reference PNM/12/13-154). Enquiring participants were sent the information sheet and invited to an assessment. Axis I psychiatric diagnoses were assessed with the MINI (Sheehan et al. 1998). Those that were assessed as eligible and agreed to participate gave written informed consent. Eligible participants were randomly assigned to MBCT (*n* = 32) or self-help (*n* = 33). Self-report questionnaires were completed pre-intervention, at the end of the 8-week MBCT intervention, and at 10-week post-intervention. Participants were sent the questionnaire pack electronically at the appropriate time point by the study’s research assistant. Participants were given approximately 1 week to respond with the completed measures. If questionnaires were not received within this time, participants were contacted by the research assistant via phone or email, on a maximum of two occasions, to prompt completion.

#### Pure Self-Help Intervention

Self-help guidance was provided in the form of a 50-page self-help booklet written by the authors for this study and specifically for use within a pure self-help format. The second author has considerable experience in working with perfectionist individuals in clinical settings. The content was based on existing cognitive behavioural approaches to perfectionism (e.g. Shafran et al. 2010) but was written specifically for students, and aimed to be a concise, readable and engaging booklet. Feedback about the booklet from perfectionist individuals, including students, was elicited as part of the refinement process. The booklet used the same CBT model of perfectionism as the adapted MBCT (with the MBCT intervention utilising some of the booklets in hand-out form to support psycho-education and discussion of perfectionism).

The booklet described how perfectionism can affect the way we think, act and feel, and outlined a CBT maintenance model. This was followed by sections aiming to help participants overcome unhelpful aspects of their perfectionism by addressing perfectionist thinking and behaviours, recognising strengths and creating a balanced life. Exercises were included throughout the booklet to encourage the application of information and learning to participants own individual circumstances (e.g. identifying one’s unhelpful thoughts and behaviours including ‘safety’ behaviours). The exercises were suggestions based on standard cognitive behavioural techniques such as thought records and behavioural experiments. Vignettes and examples of perfectionist individual’s thinking processes and behaviours, and how these could be addressed, were provided throughout. Participants were sent an electronic or hard copy of the booklet with a suggestion to regularly work through it, and encouraged to contact the researchers with any questions.

#### MBCT Intervention

The structure and practices were adapted from the MBCT course for recurrent depression (Segal et al. 2002). There were eight weekly 2-hour sessions, and participants were invited to engage in home practice, with the use of recordings of mindfulness exercises. Sessions consisted of mindfulness meditation practices, enquiry and the opportunity to discuss home practice, any obstacles or difficulties. As in standard MBCT, sessions one to four focused on helping participants learn to pay attention, and sessions five to eight on learning to handle negative thoughts or feelings. The within and between-session content of the programme was adapted so that psycho-educative and cognitive components were consistent with cognitive behavioural approaches for perfectionism. In line with this, participants were provided with weekly hand-outs about perfectionism taken from the self-help booklet described above, and depression-specific reading from the standard MBCT programme was removed. Perfectionism was discussed every session and unlike standard MBCT, a loving-kindness meditation was included towards the end of the programme aimed at helping to address the high levels of self-criticism within this population.

More specifically, the content of sessions one to three was broadly consistent with the standard MBCT protocol, with the addition of discussion about perfectionism and hand-outs from the self-help booklet. Session four adapted the psycho-education about depression to information about perfectionism and its common features in thoughts, feelings and behaviours, while session five incorporated information on ‘rules for living’ and the fight/flight response. Session six was adapted to highlight the role of self-critical thinking and explicitly focus on developing self-compassion and kindness towards the self. In session seven, exercises were adapted to explore links between positive/negative activities and mood and the early warning signs of perfectionism, with psycho-education about recognising strengths and creating a balanced life. Session eight adapted the exercises on reviewing early warning signs and developing an action plan in order to focus on perfectionism rather than depression. Participants were offered a 10-week follow-up mindfulness session (2 hours) that included mindfulness practices and enquiry, a review of participants’ current mindfulness practice and future practice intentions.

The study took place in two different universities. Ten participants were randomised to MBCT at the University of Bath and 22 participants at King’s College London. Both groups were led by an experienced MBCT instructor (KR) who met the requirements of the Good Practice Guidelines for Teaching Mindfulness-based Courses (UK Network of Mindfulness-based Teacher Trainers 2010). The instructor was assisted in Bath by a clinical psychologist in training (KJ) and in London by a qualified clinical psychologist.

#### Allocation Strategy

Randomisation was conducted by a researcher not involved in the study. A computer-generated randomisation sequence was prepared in sealed envelopes. Blocks of two were used to ensure each intervention was balanced. These envelopes remained concealed until assignment to the groups.

### Measures

The primary outcome was perfectionism; secondary outcomes included impairment caused by perfectionism and self-reported depression, anxiety and stress. Process variables included measures of self-compassion, rumination, unhelpful beliefs about emotions, mindfulness and decentering. For each measure, higher ratings indicate higher levels of the specific construct. Previous research has demonstrated each to be reliable and valid.

Axis I psychiatric diagnoses were assessed with the Mini-International Neuropsychiatric Interview (MINI.; Sheehan et al. 1998). The MINI is a short structured diagnostic interview for psychiatric difficulties and has been validated against the Structured Clinical Interview for DSM-IV-TR Axis I (SCID-I; First et al. 2002) and the Composite International Diagnostic Interview (CIDI; Wittchen et al. 2001). The interview was completed by the first author, either face-to-face or by telephone, with all participants as part of the assessment process. The MINI has high validity, internal consistency and test-retest reliability (Sheehan et al. 1997).

#### Acceptability and Engagement

Measures of engagement for MBCT included session attendance and amount of home practice undertaken (minutes per day reported on home practice sheets). Those in the self-help group were asked about the number of exercises completed within the booklet. Participants in both groups were asked to estimate the proportion (%) of hand-outs or booklet they had read, and how useful they had found the intervention, with response options of ‘no use at all’, ‘quite useful’, ‘useful’, ‘moderately useful’ and ‘very useful’. Drop-out was also investigated as an indication of acceptability.

#### Perfectionism

The 35-item FMPS (Frost et al. 1990) is a widely used measure of perfectionism. There are six subscales: Concern over Mistakes (COM), Personal Standards (PS), Parental expectations (PE), Doubts about actions (DA), Parental Criticism (PC) and Organisation (O). The subscales internal consistency ranges from .77 to .93 and it has good concurrent validity in female undergraduates (Frost et al. 1990). In line with previous intervention research (e.g. Steele et al. 2013), only two subscales were used. The COM (e.g. “If I fail at work/school, I am a failure as a person”) scale was used as the primary outcome measure as it has items closest to the concept of clinical perfectionism. The PS scale has items such as “Other people seem to accept lower standards from themselves than I do”. Items are rated on a five-point Likert scale ranging from 1 (strongly disagree) to 5 (strongly agree). In this study, Cronbach’s alphas were acceptable (COM = .85; PS = .71). Both subscales have been shown to be sensitive to change in perfectionism outcome research.

Perfectionism was further assessed with the 12-item Clinical Perfectionism Questionnaire (CPQ) (Fairburn et al. 2003), which assesses clinical perfectionism by rating the frequency of cognitive, behavioural and affective aspects of goal setting and striving over the past month on a four-point scale from ‘not at all’ to ‘all of the time’. While not solely with students, two studies (*n* = 415) by Egan et al. (2016) provided evidence of the CPQ having good discriminant and construct validity. Cronbach’s alpha was .75.

#### Impairment

The Work and Social Adjustment Scale (WASAS) (Mundt et al. 2002) is a five-item scale assessing functional impairment in work, home management, social and private activities and relationships, which was adapted to ask about the impact of perfectionism. Responses range from ‘not at all impaired’ (0) to ‘very severely impaired’ (8). Drawing on studies with depressed participants and those with obsessive compulsive disorder (OCD), the WASAS has been found to be convergent with disorder severity, as assessed by measures of depression and OCD severity, and significantly discriminated those with moderate-severe, moderate-mild and sub-clinical levels of depression and OCD severity (Mundt et al. 2002). Cronbach’s alpha was .78.

#### Stress, Anxiety and Depression

Levels of anxiety, stress and depression were assessed using the 21-item Depression Anxiety Stress Scale (DASS) (Henry and Crawford 2005). Participants rate how much they have experienced symptoms of these difficulties over the past week—responses range from ‘did not apply to me at all’ (0) to ‘applied to me very much, or most of the time’ (3). In exploring the validity of the DASS-21 in a non-clinical sample (*n* = 1794), which was broadly representative of the adult UK population, Henry and Crawford (2005) found that the measure possessed good convergent and discriminant validity when compared with other validated measures of depression and anxiety. Cronbach’s alphas ranged from .82 to .91.

#### Mindfulness

The 39-item Five-Facet Mindfulness Questionnaire (FFMQ) (Baer et al. 2006) has five factors: observing, describing, acting with awareness, non-judging of inner experience and non-reactivity to inner experience. There are five response options from ‘never or very rarely true’ (1) to ‘very often or always true’ (5). With a student sample, Baer et al. (2006) showed that the facets of mindfulness were differentially correlated in expected ways with a variety of other variables and showed incremental validity in the prediction of psychological symptoms. Cronbach’s alphas ranged from .78 to .93. This measure has previously been used in MBCT outcome research (e.g. Rimes and Wingrove 2013).

#### Beliefs About Emotions

The 12-item Beliefs about Emotions Scale (BES) assesses beliefs about the unacceptability of experiencing and expressing negative feelings (Rimes and Chalder 2010). There are seven response options from ‘totally disagree’ (0) to ‘totally agree’ (6). A previous study found that the scale is reliable and valid in a population with chronic fatigue syndrome and healthy controls (Rimes and Chalder 2010). Cronbach’s alpha was .88.

#### Self-Compassion

The 12-item Self-Compassion Scale (SCS) assesses self-compassion (Neff 2003). Neff (2016) outlines research showing that the SCS has good convergent validity when used with therapists and couples and has good discriminate validity in relation to self-esteem and self-criticism. Responses on a five-point Likert scale range from 1 (‘almost never’) to 5 (‘almost always’). There are six subscales: self-kindness, self-judgement, common humanity, isolation, mindfulness and over-identification. Mean scores are calculated for each subscale (reverse-scored where appropriate) and added to give a total score. Cronbach’s alpha was .80.

#### Decentering

The Experiences Questionnaire (Fresco et al. 2007) is an 11-item measure of decentering, demonstrating good internal consistency (.81 to .90). Using both a student (*n* = 519) and clinical sample (*n* = 220), Fresco et al. (2007) showed evidence for the convergent and discriminant validity of a decentering factor with negative relationships with measures of depressive symptoms, rumination and behavioural inhibition and a positive relationship with a measure of behavioural approach. Participants rate how much they currently have similar experiences to those described (e.g. ‘I can actually see that I am not my thoughts’). Five response choices range from ‘never’ (0) to ‘all the time’ (4). Cronbach’s alpha was .85.

#### Rumination

The Rumination Responses Questionnaire (RRQ) (Trapnell and Campbell 1999) is a 12-item measure of rumination. Trapnell and Campbell (1999) report internal consistency coefficient estimates of 0.90 and found positive associations between rumination and markers of psychological distress. Items are rated on a five-point scale ranging from 0 (‘strongly disagree’) to 4 (‘strongly agree’). Cronbach’s alpha was .71.

### Data Analyses

Preliminary analyses tested between-group comparability on demographic variables and outcome measures. Primary analysis compared the effects of MBCT with self-help immediately following the 8-week intervention utilising univariate ANCOVAs, in which the pre-treatment score on the respective outcome variable was entered as a covariate. Normality estimation indicated adequate normality for the ANCOVAs. Corrections were not made for multiple comparisons as this was a pilot study where it was important to identify possible effects that could be investigated in subsequent larger studies.

Analyses were conducted on an intention-to-treat (ITT) sample. The conservative ITT procedure utilises data from all recruited participants providing pre- and post-intervention data, regardless of whether they completed treatment, with the last observations carried forward for missing data. Similar analyses were conducted for data at 10-week post-intervention, with the pre-treatment score again entered as a covariate. Chi-square and *t* test analyses found no significant differences between those participants who completed and those who did not complete the intervention on any demographic variable or baseline outcome/process measure (all *p* < .05).

## Results

### Acceptability and Engagement

Data on levels of attendance at MBCT sessions and percentage of the self-help booklet reported to be read by self-help participants are presented in Table 1. The 16 participants who completed the MBCT (attending > 80% of sessions) attended a mean of 7.2 sessions out of 8. Chi-square analyses comparing the proportion of MBCT and self-help participants who completed ≥ 80% of the intervention suggested that there were no significant between-group differences (*χ* ^2(^(1) = 0.3, *p* = .595).

The mean total duration of weekly formal practice over MBCT, reported at post-treatment, was 109 min (SD = 46.7). The mean number of days of formal home meditation practice per week between MBCT sessions was 3.8 (SD = 1.3). For the 24 MBCT participants for whom data was available, 14 (58.3%) participants reported reading at least 80% of session hand-outs. Remaining participants reported reading 70% (*n* = 2), 60% (*n* = 1), 30% (*n* = 1), 20% (*n* = 2) and 10% (*n* = 3). One participant completed post-intervention questionnaires but did not answer these questions relating to home practice. For the self-help group, 16 participants reported that the number of exercises completed ranged from one to nine (mean = 4.4, S.D. = 2.5). Three participants did not answer these questions.

### Perceived Usefulness of the Interventions

All MBCT completers rated the course as useful, with 50% rating it as ‘very useful’ (see Table 1). Chi-square analyses (comparing those rating each intervention as either ‘no use at all’, ‘quite useful’ or ‘useful’ with those rating it as ‘moderately useful’ or ‘very useful’) showed that there were no significant between-group differences in the usefulness ratings for those who completed the interventions (*χ* ^2(^(1) = 3.4, *p* = .067). However, there were significant group differences, favouring MBCT, when including participants who did not complete the intervention (*χ* ^2^(1) = 6.7, *p* = .010).

### Group Differences at Post-Treatment

ITT ANCOVAs found significantly lower post-treatment COM, PS, clinical perfectionism and stress in the MBCT than in the self-help group, covarying for baseline scores. There were no significant group differences in impairment in daily life, anxiety or depression. ANCOVAs with process measures showed that the MBCT group had significantly lower levels of unhelpful beliefs about emotions and rumination, and higher levels of mindfulness, self-compassion and decentering at post-treatment, in comparison with the self-help group. See Table 2 for means and standard deviations at each assessment point, and results of all ANCOVAs in Table 3.

**Table 2:** Pre, post and follow-up mean scores for MBCT and self-help group

Analysis/measure	MBCT M (SD) (*n* = 28)			Self-help M (SD) (*n* = 32)		
	Pre-treatment	Post-treatment	Follow-up	Pre-treatment	Post-treatment	Follow-up
*Intention-to-treat—*clinical outcomes						
Concern over mistakes	33.9 (5.8)	27.2 (7.2)	26.8 (7.9)	31.4 (6.4)	28.4 (7.2)	29.6 (7.3)
Clinical perfectionism	29.4 (4.7)	25.8 (4.8)	25.4 (4.3)	29.0 (5.1)	27.4 (5.2)	28.0 (5.6)
Personal standards	30.0 (3.3)	26.9 (4.8)	26.2 (5.2)	29.3 (3.7)	28.4 (4.1)	28.6 (4.3)
Daily impairment by perfectionism	19.6 (8.3)	16.1 (9.6)	15.6 (9.6)	17.2 (7.7)	16.3 (8.4)	18.2 (9.9)
Anxiety	12.9 (9.0)	10.4 (9.6)	10.1 (7.7)	13.4 (10.5)	12.1 (9.8)	11.8 (9.6)
Depression	16.1 (12.1)	12.1 (12.6)	13.2 (11.5)	14.0 (10.3)	11.6 (9.7)	13.4 (11.1)
Stress	24.0 (10.2)	17.5 (11.4)	18.8 (10.8)	21.6 (9.6)	20.3 (9.9)	21.1 (9.4)
*Process measures*						
Beliefs about emotions	47.7 (13.0)	39.2 (17.3)	38.7 (14.7)	49.5 (12.2)	46.8 (13.7)	49.7 (12.5)
Decentering	26.0 (6.7)	36.1 (8.4)	34.1 (8.0)	29.4 (5.3)	30.9 (5.8)	30.8 (5.4)
Rumination	35.2 (4.5)	29.2 (6.5)	30.2 (5.9)	33.7 (5.6)	32.7 (6.1)	32.7 (6.8)
Mindfulness	104.8 (18.4)	119.5 (19.9)	119.5 (22.9)	109.3 (17.1)	111.0 (14.5)	111.6 (14.8)
Self-compassion	2.0 (0.5)	2.7 (0.7)	2.7 (0.8)	2.3 (0.5)	2.4 (0.5)	2.5 (0.7)

**Table 3:** Results of ANCOVA investigating between-group differences, adjusting for pre-treatment questionnaire scores

Analysis/measure	Post-intervention group difference						Follow-up group difference			
				90% confidence interval					90% confidence interval	
	*β*	*F*	*Partial η2*	*Lower*	*Upper*	*β*	*F*	*Partial η2*	*Lower*	*Upper*
*Intention-to-treat—*clinical outcomes										
Concern over mistakes	− 3.18	4.8*	0.08	0.00	0.19	− 4.27	5.7*	0.09	0.01	0.22
Clinical perfectionism	− 2.00	4.3*	0.69	0.00	0.20	− 2.82	6.3*	0.10	0.01	0.23
Personal standards	− 2.12	6.4*	0.10	0.01	0.23	− 3.06	10.5**	0.16	0.04	0.29
Daily impairment by perfectionism	− 2.21	_2.0	0.03	0.00	0.14	− 4.49	5.2*	0.08	0.01	0.21
Anxiety	− 1.32	_0.6	0.01	0.00	0.09	− 1.45	_0.7	0.01	0.00	0.09
Depression	− 1.22	_0.5	0.01	0.00	0.08	− 1.57	_0.5	0.01	0.00	0.09
Stress	− 4.51	4.8*	0.08	0.00	0.20	− 3.25	_1.8	0.03	0.00	0.13
*Process measures*										
Beliefs about emotions	− 10.35	10.1**	0.15	0.03	0.29	− 10.08	10.5**	0.16	0.04	0.30
Decentering	6.66	13.3**	0.19	0.06	0.33	4.36	6.1*	0.10	0.01	0.23
Rumination	− 4.73	16.5**	0.24	0.08	0.36	− 4.83	5.5*	0.09	0.01	0.22
Mindfulness	11.93	16.5**	0.22	0.08	0.36	10.92	7.6*	0.12	0.02	0.25
Self-compassion	0.42	8.6**	0.13	0.02	0.27	0.37	_3.9	0.06	0.00	0.18

*p < 0.05, **p < 0.005

### Group Differences at 10-Week Follow-Up

ITT ANCOVAs found significantly lower post-treatment COM, PS, clinical perfectionism, and impairment in daily life in the MBCT than in the self-help group, covarying for baseline scores. There were no significant group differences in stress, anxiety, depression or self-compassion. The MBCT group had significantly lower levels of unhelpful beliefs about emotions and rumination, and higher levels of mindfulness, and decentering, in comparison with the self-help group.

Analyses for those participants who fulfilled the study requirements of attending ≥ 80% of MBCT sessions or reported reading ≥ 80% of the self-help guide were also examined. Due to small sample sizes at both post-treatment (MBCT *n* = 16; self-help *n* = 13) and 10-week follow-up (MBCT *n* = 16; self-help *n* = 9), these analyses are included as supplementary material.

### Relationship Between MBCT Home Practice and Change in Psychological Variables

Pearson’s correlations showed that greater frequency of home practice per week was significantly correlated with larger increases in self-compassion (*r*(17) = 0.51, *p* = 0.04). Frequency of home practice was not significantly correlated with changes in other outcome or process measures (all *r* < 0.25).

### Reliable and Clinically Significant Change

The extent of change on the COM, clinical perfectionism and DASS-21 subscales was calculated using Jacobson and Truax’s (1991) criteria for reliable and clinically significant change. For COM, non-clinical normative data was drawn from an adult sample (*n* = 255) (Harvey et al. 2004) and clinical normative values from Steele et al. (2013). These values led to an RCI cut-off of 20.9, with a reliability coefficient of .88, as reported in Frost et al. (1990). For clinical perfectionism calculations, normative data was drawn from a community sample by Chang and Sanna (2012) (*n* = 243) and clinical normative values from Riley et al. (2007) (*n* = 20). On the basis of these values, the RCI cut-off was calculated as 30.59, with a reliability coefficient of .83, as reported by Chang and Sanna. For the DASS subscales, non-clinical normative values from an adult sample (*n* = 497) (Crawford et al. 2011) and clinical normative values from a sample of outpatients with depression and/or anxiety (*n* = 258) (Antony et al. 1998) were utilised. This led to cut-off values for clinically significant change of depression = 7.82, anxiety = 4.04 and stress = 7.59. Henry and Crawford’s reliability coefficients for each DASS subscale were adopted: depression = .88, anxiety = .82 and stress = .90. Thomas and Truax’s (2008) recommended categories of change were then used: recovered (reliable and clinically significant change), improved (reliable change without significant clinical change), same (no change) and deteriorated (reliable change with worsening symptoms). See Table 4.

**Table 4:** Number of participants meeting criterion for change in intention-to-treat sample

Measures	MBCT, *n* (%)				Self-help, *n* (%)			
	Recovered	Improved	Same	Deteriorated	Recovered	Improved	Same	Deteriorated
Post-treatment								
(MBCT = 28; Self-help = 32)								
Concern over mistakes*	6 (21)	12 (43)	10 (36)	0 (0)	4 (13)	8 (25)	20 (63)	0 (0)
Clinical perfectionism	8 (29)	0 (0)	20 (71)	0 (0)	4 (13)	0 (0)	28 (88)	0 (0)
DASS depression*	10 (36)	4 (14)	11 (39)	3 (11)	3 (9)	5 (16)	22 (69)	2 (6)
DASS anxiety	3 (11)	3 (11)	22 (79)	0 (0)	3 (9)	2 (6)	26 (81)	1 (3)
DASS stress*	5 (18)	10 (36)	10 (36)	3 (11)	3 (9)	3 (9)	24 (75)	2 (6)
10-week follow-up								
Concern over mistakes	5 (18)	11 (39)	12 (43)	0 (0)	1 (3)	0 (0)	18 (56)	4 (13)
Clinical perfectionism*	10 (36)	0 (0)	18 (64)	0 (0)	4 (13)	0 (0)	27 (84)	1 (3)
DASS depression	7 (25)	5 (18)	12 (43)	4 (14)	6 (19)	3 (9)	18 (56)	5 (16)
DASS anxiety	3 (11)	5 (18)	18 (64)	2 (7)	3 (9)	4 (13)	22 (69)	3 (9)
DASS stress*	3 (11)	9 (32)	14 (50)	2 (7)	0 (0)	6 (19)	22 (69)	4 (13)

*Significant group difference for reliable change

Chi-square analyses were undertaken to compare the two groups with regard to reliable change (i.e. ‘improved or recovered’ versus ‘same or deteriorated’). If more than 20% of the cells had an expected cell count of less than five, Fisher’s exact tests were undertaken instead. At post-treatment, a greater proportion of the MBCT group than the self-help group had shown reliable change on the COM, DASS depression and DASS stress (*p* values < 0.05); there was no group difference for clinical perfectionism or DASS anxiety. At 10-week follow-up, relative to pre-treatment scores, a greater proportion of the MBCT group than the self-help group showed benefits in clinical perfectionism and DASS stress (all *p* values < 0.05) but there were no significant group differences for COM, DASS anxiety or depression.

### Mechanisms of Change

As significant differences were observed across all process measures between pre- and post-intervention, mediational analysis assessed whether changes in perfectionism were due to changes in these hypothesised mechanisms. The bootstrapping method was used to investigate mediation, as advocated by Preacher and Hayes (2008). With this approach, mediation is investigated by directly testing the significance of the indirect effects of the independent variable (IV) on the dependent variable (DV) through a mediator (M). Bootstrapping is a nonparametric resampling procedure that involves repeatedly sampling from the data set and estimating the indirect effect in each resampled data set. By repeating this process 5000 times, 95% confidence intervals are constructed for the indirect effect. This method allows multiple mediators to be investigated, indicating the individual effects of each mediator, controlling for the other. Indirect effects were considered significant when the bias-corrected and accelerated confidence intervals did not include zero.

Mediation was investigated by deriving 95% CI for the indirect effect of group (MBCT versus self-help) via the hypothesised mediators (change in mindfulness, self-compassion, unhelpful beliefs about emotions, decentering and rumination from pre- to post-intervention) on change in COM and clinical perfectionism. Separate mediation models were run for the two perfectionism measures; see Table 5. Results indicated that change in self-compassion significantly mediated the relationship between group (MBCT versus self-help) and changes in clinical perfectionism scores.

**Table 5:** Summary of multiple mediator model (5000 bootstraps) for changes in COM and clinical perfectionism from pre- to post-intervention

Independent variable	Mediating variable	Dependent variable	Effect of IV on M		Effect of M on DV		Direct effect		Indirect effect				Total effect	
IV	M	DV	(a)		(b)		(c)		(a x b)			95% CI		
			*Coeff*	*p*	*Coeff*	*p*	*Coeff*	*p*	*Coeff*	*p*	*Lower*	*Upper*	*Coeff*	*p*
Group	Decentering	COM	− 8.73	.0000	0.07	.703	− 1.0126	.021	− 0.6208	.687	− 4.11	2.67	6.19	.021
	Rumination		4.9	.0001	0.26	.459			1.281	.439	− 3.13	5.95		
	Mindfulness		− 13.13	.0001	− 0.09	.460			1.209	.440	− 3.10	4.74		
	Self-compassion		− 0.53	.0006	− 6.57	.017			3.505	.034	− 0.12	9.28		
	Beliefs about													
	emotions		8.29	.0042	0.22	.058			1.828	.091	− 0.03	4.90		
Group	Decentering	Clinical	− 8.73	.0000	0.05	.484	0.09	.933	− 0.58	.384	− 2.82	0.99	2.15	.044
	Rumination	perfectionism	4.9	.0001	− 0.01	.950			− 0.05	.947	− 2.01	1.72		
	Mindfulness		− 13.13	.0001	− 0.04	.413			0.44	.506	− 1.60	2.56		
	Self-compassion		− 0.53	.0006	− 3.79	.002			1.98	.012	0.27	4.35		
	Beliefs about													
	emotions		8.29	.0042	0.04	.429			0.26	.014	1.44	1.44		

## Discussion

We compared the acceptability and impact of an adapted MBCT intervention with a minimal treatment control condition (a self-help CBT psycho-educational guide) in students experiencing difficulties due to perfectionism. MBCT participants had significantly lower levels of perfectionism (concern over mistakes and personal standards), clinical perfectionism and stress at post-treatment than self-help participants, adjusting for baseline levels. These benefits in perfectionism were maintained at 10-week follow-up, at which point the MBCT group also had lower levels impairment caused by perfectionism than the self-help group. Similarly, a greater proportion of MBCT than self-help participants showed reliable change in perfectionism at post-treatment and clinical perfectionism at follow-up.

Overall, these findings suggest that the adapted MBCT shows promise as an intervention for those experiencing difficulties related to perfectionism and is more beneficial than a pure CBT self-help guide. The degree of change in the perfectionism measures is not as large as in individual CBT for perfectionism (e.g. CPQ *d* = 1.31 (Riley et al. 2007); COM *d* = 1.23 and PS *d* = 0.77 (Egan et al. 2014a, b)). However, the confidence intervals around the mean group differences in the current study are relatively large due to the small sample size. Therefore, it is possible that future research using a larger sample that allowed more precise estimates may find larger effect sizes.

At post-treatment, a greater proportion of the MBCT group than the self-help group had shown reliable change on the DASS depression and DASS stress, although there was no group difference for DASS anxiety. This mindfulness intervention had additional benefits such as reductions in unhelpful beliefs about emotions, decentering and improvements in self-compassion and mindfulness which are not typically reported in CBT intervention studies and which may have wider benefits for participants beyond their perfectionism. Furthermore, mindfulness training is currently generally popular and may be perceived by students experiencing perfectionism as being more attractive and potentially less stigmatising than attending therapy. This is particularly important as individuals experiencing difficulties with perfectionism do not typically present at clinical services seeking help for their perfectionism. The intervention is provided in a group setting, which, alongside Handley et al.’s (2015) recent trial of group CBT for perfectionism, highlights the importance of interventions that require fewer therapist resources than those provided on an individual basis. Furthermore, in many locations, mindfulness or meditation groups are available to the general public which can provide support for an ongoing mindfulness practice. This may be important for maintenance of gains.

Potential mechanisms of change were also investigated, with analyses suggesting that the MBCT group had significantly lower levels of unhelpful beliefs about emotions and rumination, and higher levels of mindfulness, self-compassion and decentering at post-treatment, in comparison with the self-help group. Of these processes, there was evidence that self-compassion was particularly important, as changes in this process were found to mediate the effect of MBCT (versus self-help) on clinical perfectionism. This is consistent with evidence of self-compassion as a mediator in MBCT for recurrent depression (Kuyken et al. 2010). However, it should be noted that mediation analyses should preferably include a mediator measured at a time point between the independent and dependent variables, so these analyses should only be regarded as exploratory. Future studies should investigate self-compassion further as a potential mediator and could also investigate whether greater emphasis on self-compassion would improve the treatment effect sizes.

Treatment completion for the MBCT was moderately good. Of those randomised to MBCT, 59% completed the course and displayed high rates of session attendance and homework completion. Those who did not complete MBCT primarily suggested that finding the time to commit to it was difficult, with many acknowledging that this was related to their perfectionism. This is consistent with evidence that despite identifying many negative consequences of perfectionism, individuals reported numerous benefits and often prefer not to change their perfectionism (Egan et al. 2013). Although MBCT required attendance at eight 2-hour sessions and daily practice, treatment engagement was better than in the self-help group, with only 13 of the 33 self-help participants reporting that they had read at least 80% of the self-help guide. While this was not statistically different, this could be a power issue. The MBCT participants may have been willing to remain engaged despite the greater time involved because of the higher perceived usefulness or early impact of this intervention compared to the self-help.

Limitations of the study include drop-outs—only 72% of participants completed pre- and post-intervention assessments. No significant differences in baseline characteristics between those who remained in the study or dropped out were found. Drop-out rates should be considered in future studies as this may affect statistical power and limit generalizability. The use of LOCF as a way to manage missing data may have introduced bias into the results and resulted in confidence intervals that are too narrow (Altman 2009), therefore per protocol analyses have also been provided as supplementary information. Participants were considered to be intervention completers if they attended at least 80% of the MBCT sessions but this is an arbitrary cut-off, and future studies could investigate the impact of a lower treatment dose, such as 50%. In addition, the psycho-educational condition was developed specifically for this study, as resources were not available to provide participants with a previously evaluated self-help book, and the follow-up time period was relatively short (10 weeks).

A strength of the study was the comparison of MBCT with an active control group (pure CBT self-help). However, as MBCT was a face-to-face group intervention, non-specific factors, such as therapist and social support or learning from the contributions of other participants, may have influenced the results. Similarly, the current study was not designed to test whether the mindfulness components of the new intervention were the reason for any differences between the two groups. A future study could compare the MBCT programme with a CBT intervention matched for both CBT content and non-specific factors. Supported self-help would be an alternative cost-effective control condition which might help match the two groups for levels of participant engagement.

In conclusion, this study suggests that MBCT shows promise as an intervention for students experiencing difficulties as a result of perfectionism. MBCT for perfectionism needs investigation in larger-scale studies. Further research could also compare MBCT and group-based CBT for perfectionism in terms of recruitment, acceptability, feasibility and effectiveness. Importantly, given the findings related to the role of self-compassion, future studies should also further investigate how change in this variable is most effectively achieved and the impact this has on levels of perfectionism and its associated psychological difficulties.

## Electronic supplementary material

ESM (DOCX 33 kb)
