# Supplementary material for: Mindfulness-Based Cognitive Therapy Versus Pure Cognitive Behavioural Self-Help for Perfectionism: a Pilot Randomised Study
Source: Mindfulness (N Y). 2017 Oct 13;9(3):801–14. doi: 10.1007/s12671-017-0817-8 (PMC5968046; doi:10.1007/s12671-017-0817-8)
Supplement: Supplementary file 1 — ESM (DOCX 33 kb) [file 12671_2017_817_MOESM1_ESM.docx]

**Mindfulness-based cognitive therapy versus pure cognitive behavioural self-help for perfectionism:**

**A pilot randomised study.**

*Supplementary Materials*

***Per Protocol Analyses***

*Data analyses*

Primary analysis compared the effects of MBCT with self-help utilising univariate ANCOVAs, in which the pre-treatment score on the respective outcome variable was entered as a covariate. Corrections were not made for multiple comparisons as this was a pilot study where it was important to identify possible effects that could be investigated in subsequent larger studies. These analyses were conducted on per-protocol (PP) samples. PP analyses included participants who attended ≥ 80% of MBCT sessions or reported reading ≥ 80% of the self-help guide. Similar analyses were conducted for ten-week follow-up.

To assess whether group differences were reflected in outcomes for individual participants, the level of clinically significant change was calculated for the primary outcome measures of perfectionism (COM and CPQ) and DASS-21 subscales using Jacobson and Truax’s (1991) criteria. Jacobson & Truax’s (1991) criteria for reliable and clinically significant change were computed for the CPQ and DASS-21 subscales. The values used for the CPQ and DASS-21 change calculations were drawn from published psychometric data. Thomas & Truax’s (2008) recommended categories of change were then used: recovered (reliable and clinically significant change), improved (reliable change without significant clinical change), same (no change) and deteriorated (reliable change with worsening symptoms).

**Results**

*Group Differences at Post-Treatment*

PP ANCOVA’s showed that there were significant group differences found in the perfectionism subscales of personal standards and doubts about action. Further analyses showed that there were no significant group differences found in concern over mistakes, clinical perfectionism, impairment in daily life, depression or anxiety. In contrast, there were significant group differences on each of the five process measures. See Table 1 for means, standard deviations, and results of all ANCOVAs.

---------------------------------------

Table 1 about here

---------------------------------------

*Group Differences at 10-week follow-up*

PP ANCOVA’s showed that the MBCT group had significantly lower impairment in daily life than the self-help group. There were no significant group differences in measures of perfectionism, anxiety, depression or stress. For process measures, the MBCT group had better outcomes than the self-help group except for self-compassion, which showed no significant group difference. See Table 2 for means, standard deviations, and results of all ANCOVAs.

---------------------------------------

Table 2 about here

---------------------------------------

*Clinically significant individual change*

Using PP samples to calculate clinically significant improvement in COM, 14 (87%) MBCT participants and 9 (69%) self-help participants were clinically significantly improved at post-treatment. At 10-week follow-up, 14 (87%) MBCT and 4 (44%) self-help participants met these criteria. For clinical perfectionism, 5 (31%) MBCT participants and 1 (8%) self-help participant were clinically significantly improved at this point. At 10-week follow-up, 9 (56%) MBCT and 1 (11%) self-help participant met these criteria.

For the PP sample, more MBCT than self-help participants achieved benefits (i.e. improved or recovered) across all outcomes at post-treatment, with similar findings at 10-week follow-up. However, between-group differences using the Fisher’s exact test were significant for stress (p<.05) at post-treatment and clinical perfectionism at 10-week follow-up (p<.05) (see Table 3).

---------------------------------------

Table 3 about here

---------------------------------------

Table 1. Post-treatment: *Mean scores, standard deviations and results of ANCOVA investigating between-group differences, adjusting for pre-treatment questionnaire scores.*

|  | MBCT M (SD) (*n* = 16) | | |  | Self-help M (SD) (*n* = 13) | | |  | Group Difference | | |
| --- | --- | --- | --- | --- | --- | --- | --- | --- | --- | --- | --- |
| Analysis/Measure | Pre-treatment | Post-treatment | |  | Pre-treatment | Post-treatment | |  | *F* | *p* | *Partial η2* |
| *Clinical Outcomes* |  | | |  |  | | |  |  |  |  |
| Concern over mistakes | 33.7 (5.8) | | 25.8 (6.6) |  | 31.3 (5.9) | | 26.2 (6.4) |  | 1.2 | 0.276 | 0.03 |
| Clinical Perfectionism | 30.4 (4.9) | | 25.8 (5.3) |  | 28.7 (5.7) | | 26.9 (5.7) |  | 2.3 | 0.141 | 0.08 |
| Personal Standards | 29.9 (3.3) | | 26.4 (4.9) |  | 29.5 (3.7) | | 28.0 (4.2) |  | 10.5 | 0.003 | 0.29 |
| Doubts about Action | 16.8 (2.3) | | 15.0 (3.8) |  | 15.9 (3.1) | | 14.8 (2.1) |  | 9.1 | 0.006 | 0.26 |
| Daily impairment by perfectionism | 19.0 (8.7) | | 15.8 (10.5) |  | 16.5 (8.1) | | 16.1 (8.7) |  | 1.2 | 0.289 | 0.04 |
| Anxiety | 13.9 (9.7) | | 10.5 (11.6) |  | 11.4 (12.2) | | 10.0 (11.3) |  | 0.2 | 0.697 | 0.01 |
| Depression | 15.3 (12.9) | | 9.0 (12.1) |  | 16.1 (9.1) | | 10.2 (8.0) |  | 0.0 | 0.830 | <0.01 |
| Stress | 24.9 (10.5) | | 14.8 (11.6) |  | 20.9 (10.6) | | 18.6 (11.2) |  | 3.6 | 0.068 | 0.12 |
| *Process Measures* |  | |  |  |  | |  |  |  |  |  |
| Beliefs about emotions | 60.9 (14.5) | | 48.2 (18.4) |  | 55.5 (11.6) | | 56.9 (11.4) |  | 6.6 | 0.016 | 0.20 |
| Decentering | 36.3 (3.7) | | 27.8 (6.0) |  | 32.8 (4.5) | | 31.8 (5.4) |  | 15.4 | 0.001 | 0.37 |
| Rumination | 42.2 (5.0) | | 30.3 (9.3) |  | 37.7 (7.4) | | 36.5 (8.6) |  | 20.4 | 0.001 | 0.44 |
| Mindfulness | 104.7 (19.1) | | 127.8 (18.8) |  | 114.5 (15.2) | | 115.8 (14.1) |  | 19.1 | 0.001 | 0.42 |
| Self-compassion | 2.0 (0.5) | | 2.9 (0.7) |  | 2.4 (0.6) | | 2.6 (0.5) |  | 4.8 | 0.038 | 0.16 |

Table 2. Ten-week follow-up: *Mean scores, standard deviations and results of ANCOVA investigating between-group differences at 10-week follow-up, adjusting for pre-treatment questionnaire scores.*

|  | MBCT M (SD) (*n* = 16) | | |  | Self-help M (SD) (*n* = 9) | | | |  | | Group Difference | | | | | |  |
| --- | --- | --- | --- | --- | --- | --- | --- | --- | --- | --- | --- | --- | --- | --- | --- | --- | --- |
| Analysis/Measure | Pre-treatment | Post-treatment | Follow-Up |  | Pre-treatment | Post-treatment | Follow-Up | |  | | *F* | | *p* | | *Partial η2* | |  |
| **Per-protocol** |  |  |  |  |  |  | |  | |  | |  | |  | |  | |
| *Clinical Outcomes* |  |  |  |  |  |  | |  | |  | |  | |  | |  | |
| Concern over mistakes | 35.1 (5.8) | 25.2 (6.9) | 24.6 (8.2) |  | 30.5 (6.1) | 24.9 (5.4) | | 27.3 (6.3) | |  | | 2.7 | | 0.118 | | 0.103 | |
| Clinical Perfectionism | 30.4 (4.9) | 25.8 (5.3) | 24.6 (4.3) |  | 29.1 (5.6) | 27.0 (5.8) | | 27.9 (5.8) | |  | | 3.8 | | 0.064 | | 0.148 | |
| Personal Standards | 29.8 (3.3) | 26.2 (4.8) | 24.6 (4.6) |  | 29.5 (3.7) | 28.0 (4.2) | | 28.1 (4.6) | |  | | 3.7 | | 0.066 | | 0.139 | |
| Doubts about Action | 16.7 (2.2) | 14.9 (3.7) | 14.1 (3.2) |  | 15.9 (3.1) | 14.8 (2.1) | | 15.3 (2.4) | |  | | 3.0 | | 0.101 | | 0.113 | |
| Daily impairment by perfectionism | 19.0 (8.7) | 15.8 (10.5) | 13.9 (9.5) |  | 18.9 (7.6) | 18.1 (7.2) | | 24.8 (8.5) | |  | | 11.6 | | 0.003 | | 0.345 | |
| Anxiety | 13.9 (9.7) | 10.5 (11.6) | 9.5 (8.7) |  | 10.9 (11.9) | 10.0 (12.2) | | 8.4 (10.4) | |  | | 0.0 | | 0.958 | | 0.001 | |
| Depression | 15.3 (12.9) | 9.0 (12.1) | 10.5 (10.9) |  | 15.5 (8.0) | 9.6 (8.0) | | 10.4 (10.9) | |  | | 0.0 | | 0.956 | | 0.001 | |
| Stress | 24.9 (10.5) | 14.8 (11.6) | 17.3 (10.6) |  | 20.2 (10.1) | 17.3 (11.6) | | 18.7 (9.4) | |  | | 0.2 | | 0.68 | | 0.008 | |
| *Process Measures* |  |  |  |  |  |  | |  | |  | |  | |  | |  | |
| Beliefs about emotions | 60.9 (14.5) | 48.2 (18.4) | 45.7 (15.6) |  | 52.0 (10.2) | 56.3 (10.3) | | 57.1 (10.9) | |  | | 6.1 | | 0.022 | | 0.216 | |
| Decentering | 24.9 (7.3) | 38.8 (8.9) | 37.1 (8.3) |  | 31.6 (6.7) | 31.9 (4.0) | | 32.1 (4.5) | |  | | 4.9 | | 0.038 | | 0.181 | |
| Rumination | 36.2 (3.6) | 28.2 (6.1) | 28.8 (6.1) |  | 32.8 (4.6) | 31.8 (5.4) | | 32.8 (7.0) | |  | | 4.3 | | 0.050 | | 0.157 | |
| Mindfulness | 104.7 (19.1) | 127.8 (18.8) | 128.9 (23.1) |  | 118.0 (16.4) | 119.2 (11.8) | | 118.4 (14.0) | |  | | 5.9 | | 0.023 | | 0.213 | |
| Self-compassion | 2.0 (0.5) | 2.9 (0.7) | 3.1 (0.7) |  | 2.5 (0.6) | 2.5 (0.5) | | 2.7 (0.6) | |  | | 2.1 | | 0.158 | | 0.089 | |
|  |  | | |  |  | | | | |  | |  | | | | | |
|  |  | | |  |  | | | | |  | |  | | | | | |
|  |  | | |  |  | | | | |  | |  | | | | | |

Table 3. *Number of participants meeting criterion for change in per-protocol sample.*

|  |  | MBCT, *n* (%) | | | |  | Self-Help, *n* (%) | | | |
| --- | --- | --- | --- | --- | --- | --- | --- | --- | --- | --- |
| Measures |  | Recovered | Improved | Same | Deteriorated |  | Recovered | Improved | Same | Deteriorated |
| **Post-Treatment**  **(MBCT=16; Self-help=13)** |  |  |  |  |  |  |  |  |  |  |
| Concern Over Mistakes |  | 5 (31) | 9 (56) | 2 (13) | 0 (0) |  | 2 (15) | 7 (54) | 4 (31) | 0 (0) |
| Clinical perfectionism |  | 5 (31) | 0 (0) | 11 (69) | 0 (0) |  | 1 (8) | 0 (0) | 12 (92) | 0 (0) |
| DASS Depression |  | 7 (44) | 3 (19) | 5 (31) | 1 (6) |  | 2 (15) | 4 (31) | 6 (46) | 1 (8) |
| DASS Anxiety |  | 3 (19) | 2 (13) | 11 (69) | 0 (0) |  | 2 (15) | 1 (8) | 9 (69) | 1 (8) |
| DASS Stress* |  | 4 (25) | 8 (50) | 2 (13) | 2 (13) |  | 2 (15) | 2 (15) | 7 (54) | 2 (15) |
| **10-week Follow-Up** |  |  |  |  |  |  |  |  |  |  |
| Concern Over Mistakes |  | 5 (31) | 9 (56) | 2 (13) | 0 (0) |  | 0 (0) | 4 (44) | 4 (44) | 1 (11) |
| Clinical perfectionism* |  | 9 (56) | 0 (0) | 7 (44) | 0 (0) |  | 1 (11) | 0 (0) | 8 (89) | 0 (0) |
| DASS Depression |  | 7 (44) | 4 (25) | 3 (19) | 2 (12) |  | 5 (56) | 1 (11) | 2 (22) | 1 (11) |
| DASS Anxiety |  | 3 (19) | 4 (25) | 7 (44) | 2 (13) |  | 1 (11) | 2 (22) | 5 (56) | 1 (11) |
| DASS Stress |  | 2 (13) | 6 (38) | 7 (44) | 1 (6) |  | 0 (0) | 3 (33) | 5 (56) | 1 (11) |
|  |  |  |  |  |  |  |  |  |  |  |

*Significant group difference for reliable change
